# A Multicenter, Randomized, Controlled Clinical Trial on the Efficacy and Safety of Eucalyptol, Limonene, and Pinene Enteric Capsules in the Treatment of Chronic Rhinosinusitis With Nasal Polyps Postoperatively

**DOI:** 10.1002/clt2.70123

**Published:** 2025-12-01

**Authors:** Yutong Sima, Jianfeng Liu, Jinfeng Liu, Gang Zheng, Yi Yang, Xiaolin Peng, Yan Qi, Xiaowei Wang, Yu Zhao, Yanjun Wang, Penglong Zhao, Jinming Zhao, Yuan Zhang, Ming Zheng, Chengyao Liu, Xiaohong Song, Yi Dong, Xia Gong, Wei Lv, Zhenlin Wang, Xiangdong Wang, Luo Zhang

**Affiliations:** ^1^ Department of Otolaryngology Head and Neck Surgery Beijing Tongren Hospital Capital Medical University Beijing China; ^2^ Beijing Laboratory of Allergic Diseases, Beijing Municipal Education Commission and Beijing Key Laboratory of New Medicine and Diagnostic Technology Research for Nasal Disease Beijing Institute of Otolaryngology Beijing China; ^3^ Department of Otorhinolaryngology‐Head and Neck Surgery China‐Japan Friendship Hospital Beijing China; ^4^ Department of Otorhinolaryngology Head and Neck Surgery Beijing Chaoyang Hospital Capital Medical University Beijing China; ^5^ Department of Otolaryngology The First People's Hospital of Wenling Wenling Zhejiang China; ^6^ Department of Otorhinolaryngology Beijing Hospital National Center of Gerontology Institute of Geriatric Medicine Chinese Academy of Medical Sciences Beijing China; ^7^ Department of Otorhinolaryngology Head and Neck Surgery Skull Base Surgery Center Xuanwu Hospital Capital Medical University Beijing China; ^8^ Department of Otolaryngology‐Head and Neck Surgery Peking Union Medical College Hospital Chinese Academy of Medical Sciences Peking Union Medical College Beijing China; ^9^ Department of Otolaryngology Beijing You'an Hospital Capital Medical University Beijing China; ^10^ Department of Allergy Beijing Tongren Hospital Capital Medical University Beijing China; ^11^ Research Unit of Diagnosis and Treatment of Chronic Nasal Diseases Chinese Academy of Medical Sciences Beijing China

**Keywords:** chronic rhinosinusitis with nasal polyps, eucalyptol limonene and pinene, endoscopic sinus surgery, inflammatory, saccharin test

## Abstract

**Background:**

Eucalyptol, limonene, and pinene enteric capsules (ELP) are typical mucoactive drugs used in chronic rhinosinusitis with nasal polyps (CRSwNP). However, reliable evidence regarding its efficacy in this population remains limited. This study aimed to evaluate the clinical efficacy and safety of ELP, particularly on nasal ciliary function and inflammatory biomarkers.

**Methods:**

This prospective, randomized, controlled, multicenter clinical study enrolled CRSwNP patients who underwent endoscopic sinus surgery (ESS). Participants were randomly assigned at a 1:2 ratio to the intranasal corticosteroid (INCS) group or the ELP + INCS group. Clinical outcomes were assessed over 12 weeks using sinus computed tomography, nasal endoscopy, saccharin tests (STs) and the 22‐item sinonasal outcome tests (SNOT‐22). Local inflammation in nasal secretions were quantified via a Luminex assay, and adverse effects (AEs) was monitored throughout the study period.

**Results:**

A total of 174 CRSwNP patients were included in the final analysis. Compared to the INCS group, the ELP + INCS group demonstrated significantly greater improvements in Lund‐Mackay score (LMS) and Lund‐Kennedy score (LKS) (*p* = 0.029 and 0.025, respectively). A higher proportion of patients in the ELP + INCS group also showed improvement in the runny nose score (weeks 8 and 12), cough score (week 4), and frustration score (week 8). Furthermore, STT was significantly shorter in the ELP + INCS group at week 4 (*p* = 0.015). Subgroup analysis revealed that the ELP + INCS group had significantly lower concentrations of granulocyte‐macrophage colony‐stimulating factor (GM‐CSF), interleukin (IL)‐4, granulocyte colony‐stimulating factor (G‐CSF) and thrombopoietin (Tpo) compared to the INCS group. No significant difference was observed in the incidence of AEs between the two groups.

**Conclusion:**

The combination of ELP and INCS represents an effective and well‐tolerated treatment strategy for patients with CRSwNP following endoscopic sinus surgery.

**Trial Registration:**

The trial was registered with the Chinese Clinical Trial Registry (www.chictr.org.cn; registration number ChiCTR2200055224)

## Graphical abstract

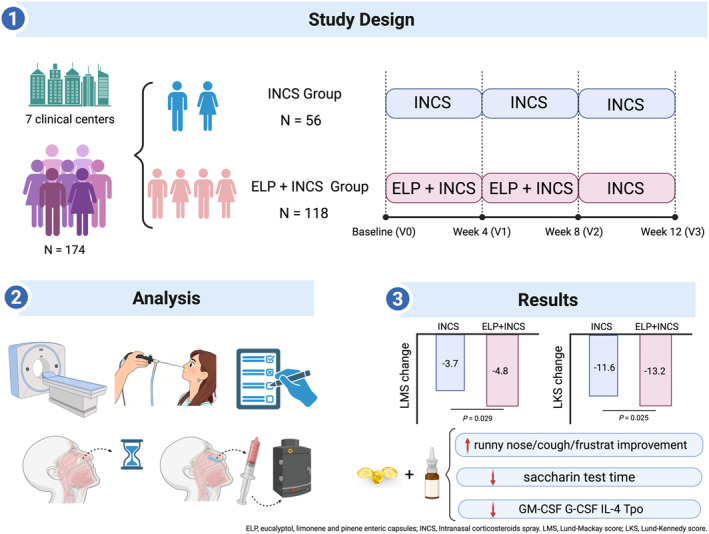

## 1 Introduction

Chronic rhinosinusitis (CRS) is a prevalent upper airway inflammatory disease that affects approximately 2%–8% of the general population in China [1]. The condition manifests with symptoms such as nasal congestion, nasal discharge, facial pain, headache and loss of smell, which substantially compromise patients' quality of life [2, 3]. Based on endoscopic finding, CRS is categorized into CRS without nasal polyps (CRSsNP) or CRS with nasal polyps (CRSwNP). Compared to CRSsNP, CRSwNP tends to have more severe clinical symptoms, experience more comorbidities, and higher rates of postoperative recurrence [4]. Moreover, The disease also imposes a considerable economic burden; a US survey revealed that approximately $8.3 billion is spent annually on CRS treatment, with most of it being spent on prescription medications [5]. Consequently, there remains a pressing need for more effective treatment strategies for CRSwNP.

CRS is a heterogeneous disease and can be divided into type 2 CRS and non‐type 2 CRS based on distinct inflammatory endotype. These inflammatory patterns directly influence symptom severity and disease prognosis. Beyond inflammation and phenotype variation, disease progression is further modulated by impaired mucociliary clearance (MCC), sinus microbiota biofilm formation, and disruption of the epithelial barrier [6, 7, 8]. In particular, recent evidence highlights that epithelial barrier damage and ciliary dysfunction exacerbate chronic inflammation and infection, leading to sinus tissue injury and remodeling. This pathophysiological insight has shifted the disease paradigm from a focus on sinus ventilation and drainage obstruction toward a mucosal‐centric model [9].

Consequently, CRS management aims to control local inflammation, restore MCC, promote sinus drainage, and enhance topical medicine delivery [10, 11]. The first‐line standardized treatment for CRS is nasal saline irrigation and corticosteroids. For type 2 CRS, biological agents (including dupilumab, omalizumab, and mepolizumab) are increasingly used alongside standard medicines. When medical therapy fails to control patient's symptoms, endoscopic sinus surgery (ESS) is indicated to remove inflamed tissue and reestablish sinus ventilation [10, 11]. Nevertheless, postoperative challenges often include persistent secretions and impaired ciliary function. Thus, adjunctive use of mucoactive agents is recommended to regulate mucus properties, enhance mucociliary transport, and mitigate airway inflammation [11]. Despite their potential clinical evidence supporting mucoactive drugs in CRSwNP remains limited.

Eucalyptol, limonene, and pinene enteric capsules (ELP) are typical mucoactive drugs derived from extracts from plants in the myrtaceae, ruaceae, and pinaceae families, which constitute a well‐established substance for treating acute and chronic rhinosinusitis. By maintaining the structural integrity and beating of cilia, [12, 13, 14] ELP was shown to increase the MCC function of ciliated cells in rats with acute exacerbation of chronic obstructive pulmonary disease [15]. It also reduces MUC5AC expression and attenuate ciliated cell damage in the lungs of patients with COPD [12]. According to the EPOS 2020 guidelines, mucoactive drugs carry a “moderate recommendation” for CRS treatment. Although recent study suggest that ELP combined with intranasal corticosteroids (INCS) improves symptoms in CRSsNP, [16] clinical evidence regarding its efficacy in CRSwNP remains scarce.

To generate high‐quality evidence on the mucoactive benefits of ELP in patients with CRSwNP following ESS and to support its clinical practice, we conducted this randomized controlled study to explore whether INCS combined with ELP can optimize clinical outcomes.

## 2 Method

### 2.1 Study Design

This was a prospective, multicenter, randomized, controlled, open‐label study conducted between May 2022 and February 2024 across seven clinical centers in China. The study protocol was approved by the respective ethics committees of the participating clinical centers. The trial was registered with the Chinese Clinical Trial Registry (www.chictr.org.cn; registration number ChiCTR2200055224). All patients provided written informed consent for participation in this study.

### 2.2 Eligibility Criteria

Patients were eligible to participate in the study if they were diagnosed with CRSwNP on the basis of the European Position Paper on Rhinosinusitis and Nasal Polyps (EPOS) 2020 guidelines. The inclusion criteria were as follows: (1) age ≥ 18 years; (2) nasal polyp score measured by Lund‐Kennedy score (LKS) higher than 2 points; and (3) Lund‒Mackay score (LMS) higher than 10 points. The key exclusion criteria were comorbid cystic fibrosis or primary ciliary dyskinesia, invasive fungal sinusitis, acute rhinitis, eosinophilic granulomatosis with polyangiitis (EGPA) or intolerance or allergy to the experimental drugs. All patients did not receive oral corticosteroids or antibiotics in the 4 weeks prior to surgery.

### 2.3 Study Intervention

All patients enrolled in this study were required to undergo functional endoscopic sinus surgery (FESS). After surgery, the patients were randomly assigned at a 2:1 ratio to the eucalyptol, limonene, and pinene enteric capsules (ELP) + intranasal corticosteroid spray (INCS) group or INCS group. Patients in the ELP + INCS group were treated with ELP (0.3 g three times/day for 4 weeks, followed by 0.3 g twice/day for 4 weeks) (Beijing Grand Johamu Pharmaceutical Company Ltd. [Beijing, China]) combined with mometasone furoate nasal spray (NASONEX, 100 µg twice/day) for 12 weeks. Patients in the INCS group were treated only with mometasone furoate nasal spray (NASONEX, 100 µg twice/day) for 12 weeks.

### 2.4 Measurement of the Lund–Kennedy Score (LKS) and Lund–Mackay Score (LMS)

Patients in both groups received nasal endoscopic examinations every 4 weeks (at baseline, week 4, week 8, and week 12) and computed tomography (CT) examinations at baseline and week 12 postoperatively. The LKS ranges from 0 to 20 points and includes scores from 5 domains: nasal polyps, discharge, edema, scarring, and crusting. The LMS ranges from 0 to 24 and includes scores related to the frontal sinus, anterior ethmoidal cells, posterior ethmoidal cells, maxillary sinus, sphenoid sinus, and ostiomeatal complex.

### 2.5 Measurement of the 22‐Sinonasal Outcomes Test (SNOT‐22)

Patients were asked to complete the SNOT‐22 questionnaire every 4 weeks (at baseline, week 4, week 8, and week 12). The SNOT‐22 questionnaire includes 22 items related to the sinuses and quality of life. Each domain ranges from 0 to 5, where 0 represents no problems and 5 indicates that the problems are as bad as they can be, which corresponds to a total score ranging from 0 to 110.

### 2.6 Saccharin Test

The saccharin test (ST) was developed by Anderson [17]. Each patient was asked to sit in a comfortable chair, raise his or her head, and tilt the head backward. A doctor placed a saccharin tablet on the medial face of the inferior turbinate, approximately 1 cm behind its anterior end, with the aid of a nasal endoscope. At the same time, the patient was asked to swallow every 30 s until a “sweet taste” of saccharin was perceived. The research investigator recorded the time from the placement of the saccharin tablet to the patient‐reported sensation of sweetness. All enrolled patients underwent ST every 4 weeks (at baseline, week 4, week 8, and week 12).

### 2.7 Analysis of Cytokine Levels in Nasal Secretions

We obtained nasal secretions from patients in both groups at baseline and week 8. Specifically, sinus sponge packs (Medtronic Xomed, Inc) were placed in both nasal sinus for 5 min to collect nasal secretion samples [18]. NaCl solution (3 mL; 0.9%) was added to the nasal sample sponges for mobilization; the sponges were then incubated at 4°C for 2 h. The sinus sponges were subsequently inserted into syringe shafts and centrifuged for 10 min at 4°C and 1500 × g. The samples were stored at −80°C for further analysis.

Cytokines in nasal secretions were analyzed with a multiple‐cytokine array on a Luminex 200 platform (Luminex Corporation, Austin, TX, USA) with a high‐sensitivity Milliplex kit (FCSTM03‐22), including interleukin (IL)‐1α, IL‐1 β, IL‐1ra, IL‐2, IL‐4, IL‐5, IL‐6, IL‐8, IL‐10, IL‐17, tumor necrosis factor (TNF)‐α, interferon (IFN)‐γ, granulocyte colony‐stimulating factor (G‐CSF), granulocyte‐macrophage colony‐stimulating factor (GM‐CSF), CCL‐2, CCL‐3, CCL‐4, CCL‐5, and CXCL5, vascular endothelial growth factor (VEGF), basic fibroblast growth factor (FGF), and thrombopoietin (Tpo). The concentrations of total protein were determined and normalized via a BCA protein assay kit (Thermo Fisher Scientific, Rockford, Illinois, USA).

### 2.8 Statistical Analysis

This pilot study was designed to provide effect‐size estimates for future trials. All the statistical analyses were carried out with R version 4.3.2 or GraphPad Prism software. Descriptive statistics and graphs were used to summarize the data. A complete case analysis was conducted for the patients who had values for all indicators throughout the study period. Parametric continuous variables are presented as the means and standard deviations, nonparametric continuous and discrete variables are presented as the medians and interquartile ranges (IQRs), and categorical variables are presented as percentages. Comparisons between the ELP + INCS and INCS groups were analyzed via the independent‐sample *t* test for parametric continuous variables, the Mann‒Whitney *U* test or Quantile Regression for nonparametric continuous and discrete variables, and the chi‐square test or Fisher's exact test for unordered categorical variables. Wilcoxon Signed Rank Test was used to compare non‐normal continuous variable and discrete variables within group. A two‐tailed *p* value < 0.05 indicated statistical significance.

## 3 Results

### 3.1 Participant Characteristics

**FIGURE 1:**
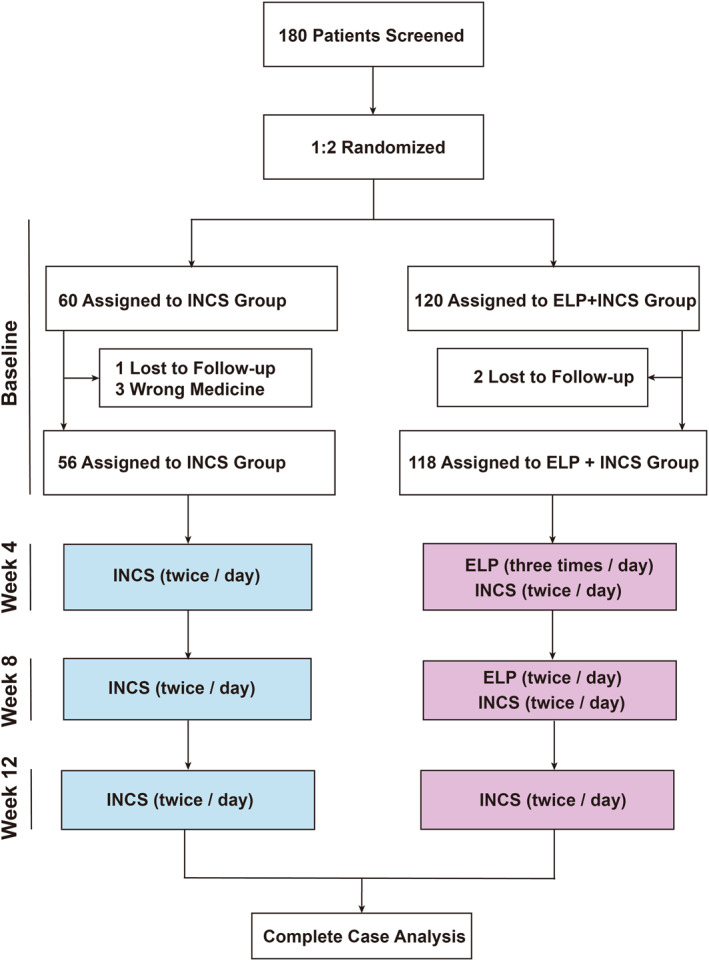
Subject disposition and composition of the analysis population.

One hundred and eighty patients were initially enrolled in this study; of these, 6 patients were excluded (2 patients in ELP + INCS group and 1 patient in INCS group were lost to follow‐up, and 3 patients in the INCS group who received oral corticosteroids or ELP therapy). Ultimately, 174 patients were included in the final analysis (Figure 1). The mean age of the enrolled patients was 45.78 ± 11.94 years, and 70.1% of them were male. Fifty‐six patients were randomized to the INCS group, and 118 patients were randomized to the ELP + INCS group. There were no differences in age, height, weight, BMI, sex distribution, comorbidities (including allergic rhinitis and asthma), history of sinus surgery, medication history before surgery, and concomitant medicines between the two groups (Table 1).

**TABLE 1:** Patient demographic characteristics.

	INCS group	ELP + INCS group	*p* value
Demographic characteristics			
Sex (male), *n* (%)	41 (73.2)	81 (68.6)	0.538^a^
Age (year), mean (SD)	45.68 (11.90)	45.83 (12.01)	0.938^b^
Height (cm), mean (SD)	169.86 (9.83)	169.61 (8.18)	0.876^b^
Weight (kg), mean (SD)	74.29 (15.38)	70.66 (11.96)	0.137^b^
BMI (kg/m^2^), mean (SD)	25.53 (3.49)	24.51 (3.17)	0.083^b^
Comorbidities, *n* (%)			
Allergic rhinitis	21 (37.5)	39 (33.1)	0.564^a^
Asthma	6 (10.7)	22 (18.6)	0.184^a^
Prior nasal polyp surgery, *n* (%)	5 (8.9)	9 (7.6)	0.771^c^
Medication history, *n* (%)			
None	31 (55.4)	60 (50.8)	0.543^d^
1–2 drugs	18 (32.1)	40 (33.9)	
3 or more drugs	7 (12.5)	18 (15.3)	
Nasal corticosteroids	14 (25.0)	30 (25.4)	0.952^b^
Concomitant medications, *n* (%)			
None	34 (60.7)	60 (50.8)	0.297^d^
1–2 drugs	11 (19.6)	32 (27.1)	
3 or more drugs	11 (19.6)	26 (22.0)	
Oral antihistamines	5 (8.9)	10 (8.5)	1.000^c^
Oral leukotriene receptor antagonists	7 (12.5)	10 (8.5)	0.403^a^

*Note:* The patients' medication history included the use of nasal corticosteroids, oral antihistamines, nasal saline irrigation, and mucolytic agents, among others. Concomitant medications included oral antihistamines, oral leukotriene receptor antagonists and others (antibiotics, nonsteroidal anti‐inflammatory drugs, etc.).

Abbreviations: ELP, eucalyptol, limonene, and pinene enteric capsules; INCS, intranasal corticosteroid; SD, standard deviation.

^a^ *t* test.

^b^ *χ* ^2^ test.

^c^ Fisher's exact test.

^d^ Mann‒Whitney *U* test.

### 3.2 Comparison of the Lund–Kennedy Score (LKS) of the ELP + INCS and INCS Groups

**FIGURE 2:**
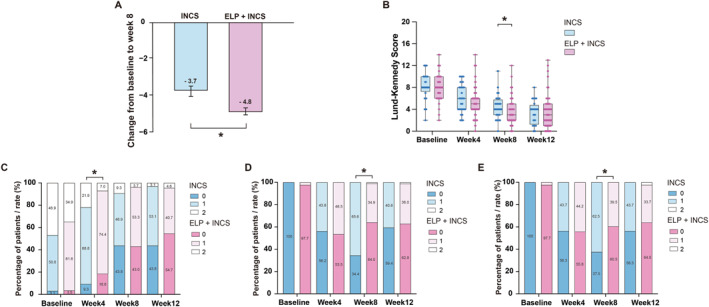
Lund–Kennedy scores of the two groups. (A) Comparisons of the change from baseline in total score (adjusted for baseline) between the ELP + INCS and INCS groups at week 8. (B) Comparisons of the total score between the ELP + INCS and INCS groups at baseline and follow‐up. (C) Comparisons of edema scores in the left sinus between the ELP + INCS and INCS groups at baseline and follow‐up. (D) Comparisons of the scarring score in the left sinus between the ELP + INCS and INCS groups at baseline and follow‐up. (E) Comparisons of the scarring score in the right sinus between the ELP + INCS and INCS groups at baseline and follow‐up. *p < 0.05.

To obtain evidence of the efficacy of ELP in treating CRSwNP, we sought to assess the conditions of the sinuses with LKS and compared them between patients treated with INCS with or without ELP. In total, 86 patients in the ELP + INCS group and 32 patients in the INCS group completed the endoscopy examination. The total LKS score at baseline and each domain score on both sides of nasal sinus did not differ between the two groups. Firstly, we compare the improvement between week 4, week 8, and week 12 and baseline. We observed the improvement in ELP + INCS group was significantly higher than that in INCS group at week 8 (adjusted median: −4.8 vs. −3.7, *p* = 0.029) (Figure 2A, and Supporting Information S1: Table S1). Subsequently, intergroup comparisons of LKS were performed at week 4, week 8 and week 12. At week 8, the total LKS in the ELP + INCS group was lower than that in the INCS group (median (IQR): 3.00 (2.00–5.00) vs. 4.00 (3.00–5.25), *p* = 0.023) (Figure 2B, Supporting Information S1: Table S2). Furthermore, each domain‐level analysis of the bilateral sinus revealed that more patients in ELP + INCS group showed lower scores in the edema score in left sinus at week 4 (*p* = 0.027; Figure 2C, and Supporting Information S1: Table S2), scarring score in the left sinus (*p* = 0.005; Figure 2D, and Supporting Information S1: Table S2), and right sinus (*p* = 0.028; Figure 2E and Supporting Information S1: Table S2) at week 8.

### 3.3 Comparison of the Lund‐Mackay Score (LMS Between the ELP + INCS and INCS Groups

**FIGURE 3:**
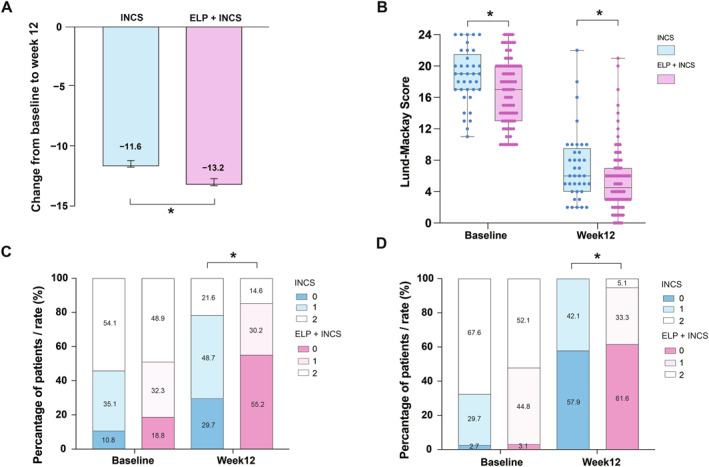
Lund‒Mackay CT scores of the two groups. (A) Comparisons of the change from baseline in total scores (adjusted for baseline) between the INCS + ELP and INCS groups at week 12. (B) Comparisons of total scores between the INCS + ELP and INCS groups at baseline and 12 weeks. (C) Comparisons of frontal sinuses in the right sinus between the INCS + ELP and INCS groups at baseline and 12 weeks. (D) Comparisons of the posterior ethmoid sinus in the right sinus between the INCS + ELP and INCS groups at baseline and 12 weeks. *p < 0.05.

The LMS is the most commonly used validated scoring system for assessing sinonasal inflammatory. Sinus CT examinations were performed on total 133 patients (96 patients in the ELP + INCS group and 37 patients in the INCS group) at baseline and 12 weeks after surgery. We compare the improvement between baseline and week 12. The improvement of LMS in ELP + INCS group was significantly higher than that in INCS group (adjusted median: −13.2 vs. −11.6, *p* = 0.025) (Figure 3A, Supporting Information S1: Table S1). Similarly, we found that the LMS in patients in the ELP + INCS group was lower than that in the INCS group both at baseline (median (IQR): 17.00 (13.00–20.00) vs. 19.00 (17.00–21.00); *p* = 0.032) and 12 weeks after surgery (median (IQR): 4.50 (3.00–7.00) vs. 6.00 (4.00–9.00); *p* = 0.016; Figure 3B, Supporting Information S1: Table S3). There was no difference in the scores of the other subdomains at baseline between the two groups. Moreover, we compared the LMS subdomains between the two groups at week 12. At the week 12 subdomain analysis, more patients in ELP + INCS group demonstrated significantly lower frontal sinus scores in right sinus (median (IQR): 0.00 (0.00–1.00) vs. 1.00 (0.00–1.00); *p* = 0.015), and the posterior ethmoid sinus scores in right sinus (median (IQR): 0.00 (0.00–1.00) vs. 1.00 (0.00–1.00); *p* = 0.042) compared to the INCS group (Figure 3C,D, Supporting Information S1: Table S3). However, with respect to the anterior ethmoid sinus, maxillary sinus, sphenoid sinus, and ostiomeatal complex, no differences between the groups were observed at week 12 (all *p* > 0.05). In conclusion, we believe that the combination of ELP and INCS demonstrates a more beneficial effect on improving sinus inflammation in patients compared to INCS alone.

### 3.4 Comparison of SNOT‐22 Scores Between the ELP + INCS and INCS Groups

**FIGURE 4:**
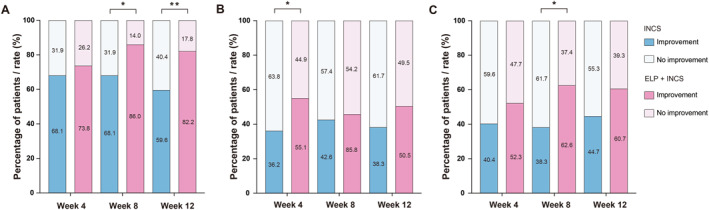
Sino‐Nasal Outcome Test (SNOT‐22) score improvement in the two groups. Comparison of the proportions of patients reporting improvements in the (A) runny nose score, (B) cough score, and (C) frustration score between the ELP + INCS and INCS groups from baseline to follow‐up. *p < 0.05, **p < 0.01.

The SNOT‐22 questionnaire directly reflects the extent of a patient's sinus‐related clinical symptoms [19]. Ultimately, 107 patients in the ELP + INCS group and 47 patients in the INCS group were included in this analysis. The baseline total SNOT‐22 and subitem scores did not differ between the ELP + INCS group and the INCS group (median (IQR): 37.00 (28.50–60.50) vs. 38.00 (22.00–51.50), *p* > 0.05; Supporting Information S1: Tables S4 and S5). Among the 22 items of the scale, a greater proportion of patients in the ELP + INCS group than in the INCS group showed improvements in the runny nose score at week 8 (86.0% vs. 68.1%, *p* = 0.010) and week 12 (82.2% vs. 59.6%, *p* = 0.003) (Figure 4A, Supporting Information S1: Table S5), whereas the cough score improvement rate was significantly greater in the ELP + INCS group than in the INCS group at week 4 (55.1% vs. 36.2%, *p* = 0.030; Figure 4B, Supporting Information S1: Table S5). Similarly, with respect to the frustration score, the improvement rate was significantly greater in the ELP + INCS group than in the INCS group at week 8 (62.6% vs. 38.3%; *p* = 0.005), but the difference did not reach the threshold for significance (*p* = 0.064) at week 12 (Figure 4C, Supporting Information S1: Table S5). With respect to the other items, no significant differences were found between the groups.

### 3.5 Comparison of Saccharin Transit Time (STT) Between the ELP + INCS and INCS Groups

**FIGURE 5:**
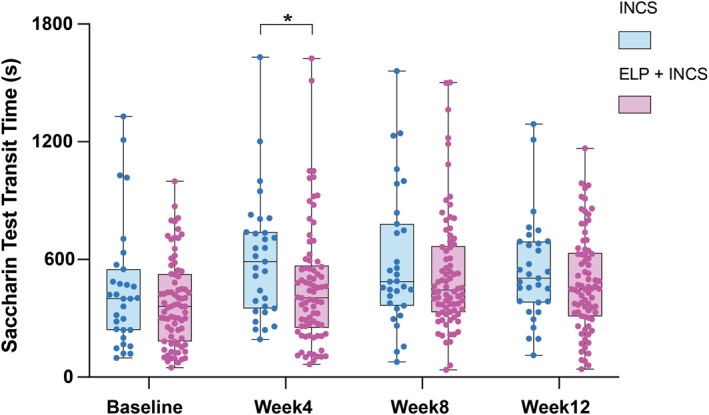
Saccharin test: transit time in the two groups. Comparisons of the saccharin transit time between the ELP + INCS and INCS groups at baseline and follow‐up. *p < 0.05.

The functionality of the MCC can be quantified with the nasal STT. In total, 78 patients in the ELP + INCS group and 31 patients in the INCS group completed the ST examination and were included in the analysis. At baseline, the STT of patients in the two groups was not different (median (IQR): 400.00 (230.00, 502.75) vs. 360.00 (202.50, 542.50)). Compared with those in the INCS group, the patients in the ELP + INCS group showed lower STT at week 4 after surgery, with median STTs of 405 s and 589 s, respectively (*p* = 0.015; Figure 5, Supporting Information S1: Table S6). However, by week 8 and week 12, the STTs of the patients in the two groups were no longer different.

### 3.6 Comparison of Nasal Secretion Cytokine Patterns Between the ELP + INCS and INCS Groups

**FIGURE 6:**
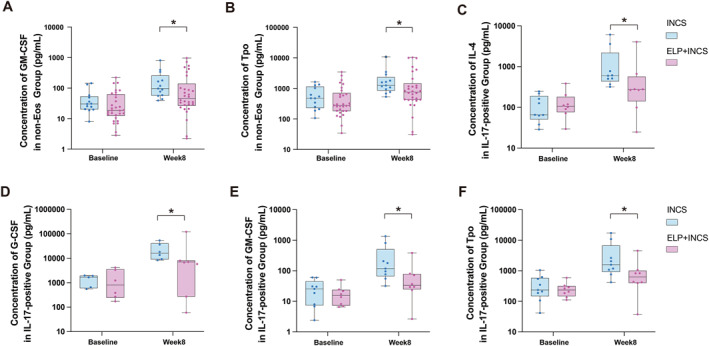
Concentrations of nasal cytokines in the two groups. Comparisons of the concentrations of (A) granulocyte macrophage‐colony‐stimulating factor (GM‐CSF) and (B) thrombopoietin (Tpo) in the non‐eosCRSwNP group; (C) IL‐4, (D) G‐CSF, (E) GM‐CSF, and (F) Tpo in the IL‐17‐positive group between INCS treatment and INCS + ELP treatment. ELP, eucalyptol, limonene, and pinene enteric capsules; INCS, intranasal corticosteroids. *p < 0.05.

The cytokine patterns of nasal secretions from 24 subjects in the INCS group and 47 subjects in the ELP + INCS group were analyzed at 2 visits (baseline and week 8). There were no differences in cytokine patterns between baseline and week 8 in either group (all *p* > 0.05, Supporting Information S1: Table S7). We further analyzed the inflammation patterns of patients with different percentages of peripheral blood eosinophils and concentrations of IL‐5 and IL‐17. We considered patients whose peripheral blood eosinophil percentage was greater than 4.27% to be in the eosinophil CRSwNP (eosCRSwNP) group (*N* = 28), and the cutoffs for IL‐5, and IL‐17 were 1900 , and 2881 pg/mL, respectively (Supporting Information S1: Table S7). In the non‐eosCRSwNP group (percentages of peripheral blood eosinophils < 4.27%, N = 43), the concentrations of GM‐CSF and Tpo were significantly lower at week 8 in the ELP + INCS group than in the INCS group (*p* = 0.042 and 0.048, respectively; Figure 6A,B, Supporting Information S1: Table S7). Additionally, the IL‐4, G‐CSF, GM‐CSF, and Tpo concentrations in the ELP + INCS group were significantly decreased among patients in the IL‐17‐positive subgroup (N = 17) (*p* = 0.036, 0.035, 0.027, and 0.046, respectively; Figure 6C,F, Supporting Information S1: Table S7). No significant differences in the two treatments were found in the eosCRSwNP group, IL‐5‐positive group, IL‐5‐negative group, or IL‐17‐negative group.

### 3.7 Adverse Effects

The overall incidence of adverse events (AEs) was comparable between the treatment groups (*p* = 0.064). A detailed listing of AEs by preferred term is provided in Table 2. The most frequently reported AE was coronavirus test positive, occurring in 6 (10.71%) patients in the INCS group and 15 (12.71%) patients in the ELP + INCS group. In the ELP + INCS group, other AEs included influenza and nasopharyngitis (each reported by 2 patients, 1.70%), as well as pyrexia and toothache (each reported by 1 patient, 0.8%). In the INCS group, 1 patient (1.79%) reported oropharyngeal pain. No severe adverse events were reported during the study. None of the observed AEs were considered related to the study treatment, and no AE led to permanent discontinuation of treatment in either group.

**TABLE 2:** Adverse events occurred during the study.

	INCS group (*N* = 56)	ELP + INCS group (*N* = 118)	*p* value
Patients with ≥ 1 adverse event	7 (12.5)	21 (17.8)	0.505^a^
Coronavirus test positive	6 (10.7)	15 (12.7)	—
Influenza	0	2 (1.7)	—
Nasopharyngitis	0	2 (1.7)	—
Oropharyngeal pain	1 (1.8)	0	—
Pyrexia	0	1 (0.8)	—
Toothache	0	1 (0.8)	—
Patients with ≥ 1 severe adverse event	0	0	—
Patients with ≥ 1 adverse event suspected to be study drug‐related	0	0	—
Patients with ≥ 1 adverse event leading to permanent discontinuation of study drug	0	0	—

*Note:* Data are presented as *n* (%).

Abbreviations: ELP, eucalyptol, limonene, and pinene enteric capsules; INCS, intranasal corticosteroids.

^a^ *χ* ^2^ test.

## 4 Discussion

Patients with CRSwNP generally have severe symptoms and poor health‐related quality of life [20]. For CRSwNP patients who undergo surgical treatment, standardized medication therapy and regular outpatient follow‐up are also required. Therefore, selecting appropriate medications to improve patients' clinical symptoms and shorten the duration of their distress requires further validation through more clinical experience. By providing the first large‐scale demonstration of ELP's clinical efficacy in CRSwNP patients, our research delivers a comprehensive evaluation of its positive effects on postoperative symptom improvement and inflammation modulation. Patients with postoperative CRSwNP in both treatment groups showed significant improvement in clinical symptoms and objective examinations (Supporting Information S1: Table S8). Notably, our study demonstrated that combination therapy with ELP and INCS resulted in superior improvement of LKS and LMS, meanwhile, associated with a greater alleviation of clinical symptom burden and a reduction in STT relative to INCS monotherapy in the early phase of postoperatively.

ELP and Myrtol standardized are both mucoactive drugs that are commonly used to treat CRS, and their main ingredients are also similar. Beyond demonstrating efficacy and safety in treating acute or chronic sinusitis, [21] meta‐analysis have also showed that Gelomyrtol improves nasal mucociliary clearance [22]. The first‐line treatment for CRSwNP, whether before or after endoscopic sinus surgery, is saline irrigation and intranasal corticosteroids. The primary goal of sinus surgery is to excise the inflammatory tissue and create larger openings in the sinuses, thereby improving the delivery of postoperative medications. INCS can improve the endoscopic scores of CRS patients and reduce recurrence rates among patients with CRSwNP [10, 11]. Other drugs, such as antibiotics, antihistamines, antileukotrienes, and mucoactive agents, can also improve clinical symptoms in CRS patients with comorbid allergic rhinitis or asthma. As confirmed by previous studies on lower airway inflammation, mucoactive agents can help eradicate inflammation [23, 24, 25, 26] and enhance mucociliary clearance, [13, 14] which may yield additional curative effects.

During the follow‐up period, patients treated with ELP + INCS showed greater improvement in the total LKS (week 8) and LMS compared with INCS group, which objective nasal endoscopic and comprehensive inflammatory evaluation offers valuable clinical practice for employing ELP as a combined postoperative therapy to INCS. Although our study demonstrated lower total LKS in the left nasal sinus in week 8 in ELP + INCS group compared to INCS group. We do not attribute this to a lateralized effect of ELP on a specific sinus, but rather to its generalized impact on the overall sinonasal.

The clinical symptoms of patients can directly impact their quality of life. Runny nose is a major clinical symptom of CRSwNP and a significant factor contributing to a poor quality of life. In this study, the runny nose symptoms improved in most patients with ELP + INCS treatment. ELP has been demonstrated to effectively promote mucus elimination; [13, 14] this effect may be related to improvements in patients' rhinorrhea symptoms. Mucus clearance from the lungs involves two steps: gas‒liquid pumping and cough‐dependent mechanisms [27]. Similarly, GeloMyrtol forte could liquefy viscous secretions, facilitate their expectoration, reduce cough frequency and reduce coughing discomfort in acute bronchitis patients [28]. A recent study reported that CRS patients at a risk of depression experience greater pain and poorer disease‐specific quality of life [29]. Patients who experience mental health problems related to CRS improve after ESS [30]. By ameliorating nasal symptoms, ELP may also positively influence patients' emotional well‐being and their perception of the disease.

The saccharin test (ST) is the most commonly used method for assessing mucociliary clearance because of its inexpensiveness, dependability, and ease of application [31, 32]. Our results revealed that at 4 weeks postoperatively, patients receiving combined ELP and INCS treatment had a significantly shorter saccharin test time (STT) compared to those receiving INCS alone. The effect of ELP on promoting ciliary function recovery has been investigated in only a limited number of studies. Li et al. reported that GeloMytrol forte increased the ciliated area in nasal epithelial cells cultured at the air‒liquid interface (ALI) for 42 days and enhanced mucin secretion after 7 days [14]. Similarly, Han et al. found that standardized myrtol improved nasal MCC in patients with CRS, which is consistent with the ciliary function improvement observed in our study [22].

Notably, our study found that the improvement in LKS showed a significant difference between the two groups at week 8, and a difference in STT was observed at week 4, but no significant differences were found at the 12‐week endpoint. The early postoperative period is critical for recovery. The ELP + INCS group showed significantly faster improvement in STT at week 4, indicating rapid restoration of mucociliary clearance essential for preventing secretion stagnation and promoting healing. Similarly, greater LKS improvement at week 8 suggested enhanced inflammation resolution and tissue repair. Accelerating recovery during this pivotal window significantly improves early symptoms and quality of life.

The degree of local inflammation in the nasal sinus can be effectively and conveniently assessed through nasal secretions. ELP has been shown to reduce the concentrations of the inflammatory cytokines TNF‐α and IL‐6 in the lungs of mice exposed to cigarette smoke, [33] which suggests a potential role in persistent airway disease inflammation. In our study, we evaluated the effect of ELP on nasal inflammation by comparing the levels of biomarkers in the nasal secretions between two treatment groups. Type 2 inflammation and eosinophilic infiltration are typical features of refractory CRSwNP [34]. In a recent study, a mixed eosinophilic and neutrophilic endotype of CRSwNP was shown to present with a more complex inflammatory endotype, presented with more severe clinical symptoms and a higher postoperative recurrence rate [35]. IL‐4 and GM‐CSF are typical type 2 inflammatory biomarkers in CRSwNP. It is interesting that the decrease was showed in non‐eosinophilic subgroup and IL‐17‐positive subgroup. We thought the cross‐interaction of this inflammation is related to the mixed endotype observed in CRSwNP patients. The reduction in local inflammation more accurately reflects the treatment efficacy, even induces long‐term effects. INCS can effectively improve nasal inflammation after ESS, [36] whereas previous studies have shown that CRSwNP with neutrophil‐dominant inflammation reduces the response to oral corticosteroid therapy [37, 38]. Our study revealed that neutrophilic inflammation biomarker G‐CSF in the IL‐17‐positive group lower in ELP +INCS group than INCS group. We thought that this may be due to the anti‐inflammatory effects of ELP, especially with respect to neutrophilic inflammation. We did not directly compare the changes in inflammatory factor levels between baseline and 8 weeks postoperatively, because the use of materials inserted after endonasal surgery could influence the local inflammatory response. Therefore, we compared the two groups and analyzed the results accordingly.

Among the commonly reported adverse effects associated with ELP, gastrointestinal symptoms are the most frequent. However, in our study, no patients reported gastrointestinal reactions. Additionally, as our research was conducted during the COVID‐19 pandemic, a number of adverse event reports were related to SARS‐CoV‐2 infection. This, however, is unrelated to ELP medication. In summary, we conclude that the combination of ELP and INCS is a safe treatment for patients with CRSwNP following endoscopic sinus surgery.

This study has several clinical implications. First, through real‐world clinical practice, the combination of ELP and INCS demonstrated a significant improvement in both LMS and LKS, and milder clinical symptoms compared INCS monotherapy for patients postoperative. Second, ELP administration was associated with a significant reduction STT during the early period, which aid in the recovery of MCC function. Third, ELP may contribute to the reduction of local nasal inflammation in patients with CRSwNP postoperatively. However, several limitations should be acknowledged. Firstly, this is an open‐label study. ELP is volatile terpenes with a characteristic and potent minty aroma, which makes effectively blinding through the creation of a matched placebo capsule extremely challenging and cost‐prohibitive. Second, the improvement in nasal mucociliary function by ELP was supported by STT data, its lacks validation through fundamental mechanistic studies. And the underlying mechanistic studies are already underway, with results expected to be published in the future. In summary, we demonstrated that the combination of ELP and INCS represents an effective and well‐tolerated treatment strategy for patients with CRSwNP following endoscopic sinus surgery, particular during the early postoperative period.

## Author Contributions

**Yutong Sima:** writing – original draft, data curation, methodology, conceptualization, writing – review and editing. **Jianfeng Liu:** writing – original draft, conceptualization. **Jinfeng Liu:** writing – original draft, conceptualization. **Gang Zheng:** writing – original draft, conceptualization. **Yi Yang:** writing – original draft, conceptualization. **Xiaolin Peng:** project administration. **Yan Qi:** project administration. **Xiaowei Wang:** project administration. **Yu Zhao:** project administration. **Yanjun Wang:** project administration. **Penglong Zhao:** project administration. **Jinming Zhao:** project administration, investigation. **Yuan Zhang:** investigation, project administration. **Ming Zheng:** project administration. **Chengyao Liu:** project administration. **Xiaohong Song:** project administration. **Yi Dong:** project administration. **Xia Gong:** project administration. **Wei Lv:** writing – review and editing, methodology, conceptualization, supervision. **Zhenlin Wang:** conceptualization, methodology, writing – review and editing, supervision. **Xiangdong Wang:** conceptualization, methodology, writing – original draft, supervision, funding acquisition. **Luo Zhang:** funding acquisition, writing – original draft, conceptualization, methodology, writing – review and editing, supervision.

## Funding

This project was supported by the Special Funds for the Construction of High‐level Public Health Technical Talents (Lingjunrencai‐01‐08 and Lingjunrencai‐02‐09), the National Natural Science Foundation of China (82000962, 82171110, and 82471137), the China International Medical Foundation (Z‐2018‐31‐2108), the National Key R&D Program of China (2022YFC2504100), the program for the Changjiang Scholars and Innovative Research Team (IRT13082), the CAMS Innovation Fund for Medical Sciences (2019‐I2M‐5‐022), the Capital's Funds for Health Improvement and Research (2022‐1‐1091), the Beijing Natural Science Foundation (7222024), the Beijing Hospitals Authority Youth Programme (QML20230201), the Public Welfare Development and Reform Pilot Project (2019‐10), and the Beijing Municipal Science & Technology Commission (Z211100002921057).

## Ethics Statement

This study was approved by the Ethics Committee of Beijing Tongren Hospital, Xuanwu Hospital, Peking Union Medical College Hospital, China‐Japan Friendship Hospital, Beijing Chaoyang Hospital, The First People's Hospital of Wenling, Beijing Hospital and the Chinese Clinical Trail Registry.

## Consent

Written informed consent was obtained from all participants. All the authors have read the final manuscript and agreed to the publication of the work.

## Conflicts of Interest

The authors declare no conflicts of interest.

## Supporting information


Supporting Information S1

## Data Availability

The corresponding authors will provide the datasets that support the study's findings on reasonable request.
