# Supplementary material for: A Multicenter, Randomized, Controlled Clinical Trial on the Efficacy and Safety of Eucalyptol, Limonene, and Pinene Enteric Capsules in the Treatment of Chronic Rhinosinusitis With Nasal Polyps Postoperatively
Source: Clin Transl Allergy. 2025 Dec 1;15(12):e70123. doi: 10.1002/clt2.70123 (PMC12669804; doi:10.1002/clt2.70123)
Supplement: Supplementary file 1 — Supporting Information S1 [file CLT2-15-e70123-s001.docx]

**Table S1.** Comparison of change from baseline between two groups.

|  | ELP + INCS group | | |  | INCS group | | |  | *P_1_* value^a^ | *P_2_* value^a^ | *P_3_* value^a^ |
| --- | --- | --- | --- | --- | --- | --- | --- | --- | --- | --- | --- |
|  | Week 4 | Week 8 | Week 12 |  | Week 4 | Week 8 | Week 12 |  |  |  |  |
| Lund–Kennedy total scores, adjusted median | -2.83 | -4.83 | -5.00 |  | -2.33 | -3.67 | -4.00 |  | 0.570 | **0.029*** | 0.259 |
| Lund–Mackay total score, adjusted median | / | / | -13.18 |  | / | / | -11.64 |  | / | / | **0.025*** |
| SNOT-22 total score, adjusted median | -24.16 | -26.00 | -29.83 |  | -20.19 | -25.60 | -28.17 |  | 0.211 | 0.868 | 0.465 |
| Saccharin transit time (STT), adjusted median | 61.93 | 81.33 | 108.15 |  | 180.00 | 159.29 | 137.98 |  | 0.128 | 0.227 | 0.560 |

INCS, intranasal corticosteroids; ELP, pinene enteric capsule; SNOT-22, the 22-Sinonasal Outcomes Test. ^a^ Quantile regression, adjusted for baseline measurement. *P_1_* value, the difference in change from baseline between two groups at week 4; *P_2_* value, the difference in change from baseline between two groups at week 8; *P_3_* value, the difference in change from baseline between two groups at week 12. * P<0.05.

**Table S2.** Comparison of Lund-Kennedy scores.

|  | INCS group | | | |  | ELP + INCS group | | | |  | *P_1_* value^a^ | *P_2_* value^a^ | *P_3_* value^a^ | *P_4_* value^a^ |
| --- | --- | --- | --- | --- | --- | --- | --- | --- | --- | --- | --- | --- | --- | --- |
|  | Baseline | Week 4 | Week 8 | Week 12 |  | Baseline | Week 4 | Week 8 | Week 12 |  |  |  |  |  |
| **Left sinus** | | | | | | | | | | | | | | |
| Polyp | 2.00  [1.00, 2.00] | 0.00  [0.00, 0.00] | 0.00  [0.00, 0.00] | 0.00  [0.00, 0.00] |  | 2.00   [1.00, 2.00] | 0.00  [0.00, 0.00] | 0.00   [0.00, 0.00] | 0.00  [0.00, 0.00] |  | 0.299 | 0.816 | 0.394 | 0.561 |
| Edema | 1.00  [1.00, 2.00] | 1.00  [1.00, 1.00] | 1.00  [0.00, 1.00] | 1.00  [0.00, 1.00] |  | 1.00  [1.00, 2.00] | 1.00  [1.00, 1.00] | 1.00  [0.00, 1.00] | 0.00  [0.00, 1.00] |  | 0.256 | **0.027*** | 0.805 | 0.361 |
| Discharge | 1.00  [1.00, 1.00] | 1.00  [0.00, 1.00] | 0.50  [0.00, 1.00] | 0.00  [0.00, 1.00] |  | 1.00  [1.00, 1.00] | 1.00  [0.00, 1.00] | 0.00  [0.00, 1.00] | 0.00  [0.00, 1.00] |  | 0.206 | 0.459 | 0.230 | 0.619 |
| Scarring | 0.00  [0.00, 0.00] | 0.00  [0.00, 1.00] | 1.00  [0.00, 1.00] | 0.00  [0.00, 1.00] |  | 0.00   [0.00, 0.00] | 0.00  [0.00, 1.00] | 0.00  [0.00, 1.00] | 0.00  [0.00, 1.00] |  | 0.394 | 0.793 | **0.005**** | 0.774 |
| Crusting | 0.00  [0.00, 0.00] | 1.00  [0.00, 1.00] | 0.00  [0.00, 1.00] | 0.00  [0.00, 0.00] |  | 0.00  [0.00, 0.00] | 1.00  [0.00, 1.00] | 0.00  [0.00, 0.00] | 0.00  [0.00, 0.00] |  | 0.220 | 0.488 | 0.282 | 0.406 |
| Total score | 4.00  [3.75, 5.00] | 3.00  [2.00, 4.00] | 2.00  [2.00, 3.00] | 2.00  [1.00, 2.25] |  | 4.00  [3.00, 5.00] | 2.00  [2.00, 3.00] | 1.00  [1.00, 2.00] | 1.00  [0.00, 2.00] |  | 0.693 | 0.076 | **0.004**** | 0.389 |
| **Right sinus** | | | | | | | | | | | | | | |
| Polyps | 2.00  [1.75, 2.00] | 0.00  [0.00, 0.00] | 0.00  [0.00, 0.00] | 0.00   [0.00, 0.00] |  | 2.00  [1.00, 2.00] | 0.00  [0.00, 0.00] | 0.00  [0.00, 0.00] | 0.00  [0.00, 0.00] |  | 0.073 | 0.302 | 0.338 | 0.995 |
| Edema | 1.00  [1.00, 2.00] | 1.00  [1.00, 1.00] | 1.00  [0.00, 1.00] | 1.00  [0.00, 1.00] |  | 1.00  [1.00, 2.00] | 1.00  [1.00, 1.00] | 1.00  [0.00, 1.00] | 1.00  [0.00, 1.00] |  | 0.396 | 0.200 | 0.840 | 0.637 |
| Discharge | 1.00  [1.00, 1.00] | 1.00  [0.00, 1.00] | 0.00  [0.00, 1.00] | 0.00  [0.00, 1.00] |  | 1.00  [1.00, 1.00] | 1.00  [0.00, 1.00] | 0.00  [0.00, 1.00] | 0.00  [0.00, 1.00] |  | 0.182 | 0.789 | 0.653 | 0.952 |
| Scarring | 0.00  [0.00, 0.00] | 0.00  [0.00, 1.00] | 1.00  [0.00, 1.00] | 0.00  [0.00, 1.00] |  | 0.00  [0.00, 0.00] | 0.00  [0.00, 1.00] | 0.00  [0.00, 1.00] | 0.00  [0.00, 1.00] |  | 0.394 | 0.969 | **0.0278*** | 0.512 |
| Crusting | 0.00  [0.00, 0.00] | 1.00  [0.00, 1.00] | 0.00  [0.00, 0.25] | 0.00  [0.00, 0.00] |  | 0.00  [0.00, 0.00] | 0.00  [0.00, 1.00] | 0.00  [0.00, 1.00] | 0.00  [0.00, 0.00] |  | 0.716 | 0.305 | 0.789 | 0.898 |
| Total score | 4.00  [3.75, 5.00] | 3.00  [2.00, 4.00] | 2.00  [1.00, 3.00] | 2.00  [1.00, 3.00] |  | 4.00  [3.00, 5.00] | 2.00  [2.00, 3.00] | 2.00  [1.00, 3.00] | 1.50  [1.00, 2.00] |  | 0.482 | 0.164 | 0.257 | 0.546 |
| **Total score** | 8.00  [7.75, 10.00] | 6.00  [4.00, 8.00] | 4.00  [3.00, 5.25] | 4.00  [1.75, 4.25] |  | 8.00  [6.25, 9.75] | 5.00  [4.00, 6.00] | 3.00  [2.00, 5.00] | 3.00  [1.00, 5.00] |  | 0.508 | 0.111 | **0.023*** | 0.414 |

Data are presented as median (IRQ). INCS, intranasal corticosteroids; ELP, pinene enteric capsule; IQR, interquartile range；^a^ Mann‒Whitney U test. *P_1_* value, the difference between two groups at baseline; *P_2_* value, the difference between two groups at week 4; *P_3_* value, the difference between two groups at week 8; *P_4._*value, the difference between two groups at week 12. * P<0.05, ** P<0.01.

**Table S3.** Comparison of Lund–Mackay scores.

|  | INCS group (N=37) | |  | ELP + INCS group (N=96) | |  | *P_1_* value^a^ | *P_2_* value^a^ |
| --- | --- | --- | --- | --- | --- | --- | --- | --- |
|  | Baseline | Week 12 |  | Baseline | Week 12 |  |  |  |
| **Left sinus** |  |  |  |  |  |  |  |  |
| Maxillary sinus,  median (IQR) | 1.00 [1.00, 2.00] | 1.00 [1.00, 1.00] |  | 1.00 [1.00, 2.00] | 1.00 [0.75, 1.00] |  | 0.723 | 0.506 |
| Anterior ethmoid sinus,  median (IQR) | 2.00 [2.00, 2.00] | 0.00 [0.00, 1.00] |  | 2.00 [1.00, 2.00] | 0.00 [0.00, 1.00] |  | 0.109 | 0.427 |
| Posterior ethmoid sinus,  median (IQR) | 2.00 [1.00, 2.00] | 1.00 [0.00, 1.00] |  | 2.00 [1.00, 2.00] | 0.00 [0.00, 1.00] |  | 0.664 | 0.116 |
| Sphenoid sinus,  median (IQR) | 1.00 [1.00, 2.00] | 0.00 [0.00, 1.00] |  | 1.00 [0.00, 1.00] | 0.00 [0.00, 1.00] |  | 0.117 | 0.052 |
| Frontal sinus,  median (IQR) | 1.00 [1.00, 2.00] | 1.00 [0.00, 1.00] |  | 1.00 [1.00, 2.00] | 0.00 [0.00, 1.00] |  | 0.279 | 0.382 |
| Ostiomeatal complex,  median (IQR) | 2.00 [2.00, 2.00] | 0.00 [0.00, 0.00] |  | 2.00 [2.00, 2.00] | 0.00 [0.00, 0.00] |  | 0.703 | 0.542 |
| Total score,  median (IQR) | 9.00 [8.00, 10.00] | 3.00 [2.00, 4.00] |  | 9.00 [7.00, 10.00] | 2.00 [1.00, 4.00] |  | 0.111 | 0.051 |
| **Right sinus** |  |  |  |  |  |  |  |  |
| Maxillary sinus,  median (IQR) | 1.00 [1.00, 2.00] | 1.00 [1.00, 1.00] |  | 1.00 [1.00, 2.00] | 1.00 [0.75, 1.00] |  | 0.139 | 0.073 |
| Anterior ethmoid sinus,  median (IQR) | 2.00 [2.00, 2.00] | 1.00 [0.00, 1.00] |  | 2.00 [1.00, 2.00] | 0.00 [0.00, 1.00] |  | 0.076 | 0.147 |
| Posterior ethmoid sinus,  median (IQR) | 2.00 [1.00, 2.00] | 1.00 [0.00, 1.00] |  | 2.00 [1.00, 2.00] | 0.00 [0.00, 1.00] |  | 0.119 | **0.042*** |
| Sphenoid sinus,  median (IQR) | 1.00 [1.00, 2.00] | 0.00 [0.00, 1.00] |  | 1.00 [0.00, 1.00] | 0.00 [0.00, 1.00] |  | **0.009**** | 0.108 |
| Frontal sinus,  median (IQR) | 2.00 [1.00, 2.00] | 1.00 [0.00, 1.00] |  | 1.00 [1.00, 2.00] | 0.00 [0.00, 1.00] |  | 0.425 | **0.015*** |
| Ostiomeatal complex,  median (IQR) | 2.00 [2.00, 2.00] | 0.00 [0.00, 0.00] |  | 2.00 [2.00, 2.00] | 0.00 [0.00, 0.00] |  | **0.043*** | 0.969 |
| Total score,  median (IQR) | 10.00 [8.00, 11.00] | 4.00 [2.00, 5.00] |  | 9.00 [7.00, 10.00] | 2.00 [1.00, 4.00] |  | **0.012*** | **0.022*** |
| **Total score, median (IQR)** | 19.00 [17.00, 21.00] | 6.00 [4.00, 9.00] |  | 17.00 [13.00, 20.00] | 4.50 [3.00, 7.00] |  | **0.032*** | **0.016*** |

INCS, intranasal corticosteroids; ELP, pinene enteric capsule; IQR, interquartile range. ^a^ Mann‒Whitney U test. *P_1_* value, the difference between two groups at baseline; *P_2_* value, the difference between two groups at week 12. * P<0.05, ** P<0.01.

**Table S4.** Comparison of total SNOT-22 scores between two groups for 12 weeks.

|  | ELP + INCS group (N=107) | INCS group (N=47) | *P* value |
| --- | --- | --- | --- |
| Baseline, median (IQR) | 38.00 [22.00, 51.50] | 37.00 [28.50, 60.50] | 0.335^a^ |
| Week 4, median (IQR) | 17.00 [7.00, 25.50] | 13.00 [7.00, 27.00] | 0.696^a^ |
| Week 8, median (IQR) | 10.00 [6.00, 18.50] | 11.00 [5.00, 21.50] | 0.959^a^ |
| Week 12, median (IQR) | 9.00 [4.00, 15.00] | 9.00 [3.50, 18.00] | 0.937^a^ |

INCS, intranasal corticosteroids; ELP, pinene enteric capsule; IQR, interquartile range. ^a^ Mann‒Whitney U test, ^b^ χ^2^ test, ^c^ Fisher’s exact test.

**Table S5.** The improvement rate of SNOT-22 during the 12 weeks.

|  | INCS group (N=47) | | |  | ELP + INCS group (N=107) | | |  | *P_1_* value | *P_2_* value | *P_3_* value |
| --- | --- | --- | --- | --- | --- | --- | --- | --- | --- | --- | --- |
|  | Week 4 | Week 8 | Week 12 |  | Week 4 | Week 8 | Week 12 |  |  |  |  |
| Need to blow nose | 35 (74.5) | 38 (80.9) | 37 (78.7) |  | 81 (75.7) | 88 (82.2) | 84 (78.5) |  | 0.870^b^ | 0.837^b^ | 0.976^b^ |
| Sneezing | 27 (57.4) | 28 (59.6) | 28 (59.6) |  | 70 (65.4) | 71 (66.4) | 79 (73.8) |  | 0.345^b^ | 0.419^b^ | 0.077^b^ |
| Runny nose | 32 (68.1) | 32 (68.1) | 28 (59.6) |  | 79 (73.8) | 92 (86.0) | 88 (82.2) |  | 0.464^b^ | **0.010**^b*^ | **0.003**^b**^ |
| Cough | 17 (36.2) | 20 (42.6) | 18 (38.3) |  | 59 (55.1) | 49 (45.8) | 54 (50.5) |  | **0.030**^b*^ | 0.710^b^ | 0.163^b^ |
| Postnasal discharge | 26 (55.3) | 29 (61.7) | 27 (57.4) |  | 48 (44.9) | 58 (54.2) | 57 (53.3) |  | 0.232^b^ | 0.388^b^ | 0.632^b^ |
| Thick nasal discharge | 28 (59.6) | 34 (72.3) | 30 (63.8) |  | 67 (62.6) | 70 (65.4) | 76 (71.0) |  | 0.721^b^ | 0.398^b^ | 0.374^b^ |
| Decreased sense of smell/taste | 29 (61.7) | 32 (68.1) | 34 (72.3) |  | 57 (53.3) | 80 (74.8) | 80 (74.8) |  | 0.332^b^ | 0.391^b^ | 0.752^b^ |
| Nasal blockage | 32 (68.1) | 37 (78.7) | 38 (80.9) |  | 85 (79.4) | 89 (83.2) | 89 (83.2) |  | 0.129^b^ | 0.509^b^ | 0.727^b^ |
| Ear fullness | 15 (31.9) | 18 (38.3) | 18 (38.3) |  | 47 (43.9) | 55 (51.4) | 50 (46.7) |  | 0.162^b^ | 0.134^b^ | 0.332^b^ |
| Dizziness | 25 (53.2) | 24 (51.1) | 27 (57.4) |  | 51 (47.7) | 60 (56.1) | 60 (56.1) |  | 0.527^b^ | 0.565^b^ | 0.874^b^ |
| Ear pain | 9 (19.1) | 9 (19.1) | 7 (14.9) |  | 27 (25.2) | 25 (23.4) | 26 (24.3) |  | 0.411^b^ | 0.561^b^ | 0.190^b^ |
| Facial pain/pressure | 18 (38.3) | 20 (42.6) | 18 (38.3) |  | 42 (39.3) | 54 (50.5) | 55 (51.4) |  | 0.911^b^ | 0.365^b^ | 0.134^b^ |
| Difficulty falling asleep | 28 (59.6) | 26 (55.3) | 28 (59.6) |  | 68 (63.6) | 62 (57.9) | 65 (60.7) |  | 0.639^b^ | 0.762^b^ | 0.891^b^ |
| Wake up at night | 25 (53.2) | 25 (53.2) | 25 (53.2) |  | 58 (54.2) | 58 (54.2) | 59 (55.1) |  | 0.907^b^ | 0.907^b^ | 0.823^b^ |
| Lack of a good night's sleep | 30 (63.8) | 31 (66.0) | 32 (68.1) |  | 61 (57.0) | 63 (58.9) | 66 (61.7) |  | 0.428^b^ | 0.407^b^ | 0.447^b^ |
| Waking up tired | 25 (53.2) | 26 (55.3) | 28 (59.6) |  | 57 (53.3) | 58 (54.2) | 65 (60.7) |  | 0.993^b^ | 0.898^b^ | 0.891^b^ |
| Fatigue | 26 (55.3) | 31 (66.0) | 33 (70.2) |  | 59 (55.1) | 66 (61.7) | 66 (61.7) |  | 0.984^b^ | 0.613^b^ | 0.309^b^ |
| Reduced productivity | 29 (61.7) | 34 (72.3) | 34 (72.3) |  | 60 (56.1) | 61 (57.0) | 65 (60.7) |  | 0.515^b^ | 0.072^b^ | 0.167^b^ |
| Reduced concentration | 27 (57.4) | 32 (68.1) | 32 (68.1) |  | 63 (58.9) | 67 (62.6) | 69 (64.5) |  | 0.868^b^ | 0.514^b^ | 0.665^b^ |
| Frustrated | 19 (40.4) | 18 (38.3) | 21 (44.7) |  | 56 (52.3) | 67 (62.6) | 65 (60.7) |  | 0.173^b^ | **0.005**^b**^ | 0.064^b^ |
| Sad | 21 (44.7) | 20 (42.6) | 21 (44.7) |  | 54 (50.5) | 62 (57.9) | 62 (57.9) |  | 0.508^b^ | 0.078^b^ | 0.128^b^ |
| Embarrassed | 21 (44.7) | 24 (51.1) | 23 (48.9) |  | 51 (47.7) | 57 (53.3) | 59 (55.1) |  | 0.733^b^ | 0.801^b^ | 0.477^b^ |
| Total score | 41 (87.2) | 43 (91.5) | 43 (91.5) |  | 93 (86.9) | 101 (94.4) | 99 (92.5) |  | 0.957^b^ | 0.495^c^ | 1.000^c^ |

Data are presented as n (%). INCS, intranasal corticosteroids; ELP, pinene enteric capsule; IQR, interquartile range. ^a^ Mann‒Whitney U test, ^b^ χ^2^ test, ^c^ Fisher’s exact test; *P_1_* value, the difference between two groups at week 4; *P_2_* value, the difference between two groups at week 8; *P_3_* value, the difference between two groups at week 12. * P<0.05, ** P<0.01.

**Table S6.** Saccharin transit time for each group and results of statistical analysis.

|  | INCS group  N=31 | ELP + INCS group  N=78 | *P* value^a^ |
| --- | --- | --- | --- |
| Baseline, median (IQR) | 400.00 [240.00, 518.50] | 360.00 [186.75, 521.25] | 0.466 |
| Week 4, median (IQR) | 589.00 [355.50, 737.50] | 405.00 [259.25, 561.25] | **0.015*** |
| Week 8, median (IQR) | 487.00 [371.00, 764.50] | 444.50 [334.25, 663.25] | 0.350 |
| Week 12, median (IQR) | 504.00 [383.50, 686.50] | 449.50 [315.25, 628.50] | 0.241 |

INCS, intranasal corticosteroids; ELP, pinene enteric capsule; IQR, interquartile range. ^a^ Mann‒Whitney U test. * P<0.05.

**Table S7.** Patterns of inflammatory factors identified in nasal secretions

|  | INCS group | ELP+INCS group | *P* value^a^ |
| --- | --- | --- | --- |
| **Non-eosCRSwNP group (N)** | 13 | 30 |  |
| GM-CSF^b^ (pg/mL, median [IQR]) | 96.56 [58.48, 178.38] | 46.22 [28.20, 98.03] | **0.042** |
| TPO (pg/mL, median [IQR]) | 1253.54 [939.08, 1796.92] | 761.13 [425.61, 1225.41] | **0.048** |
| **IL-17-positive group (N)** | 9 | 8 |  |
| IL-4 (pg/mL, median [IQR]) | 597.55 [503.79, 774.71] | 273.09 [224.96, 374.67] | **0.036** |
| G-CSF (pg/mL, median [IQR]) | 15994.91 [10389.55, 31998.09] | 7038.65 [3503.30, 8096.94] | **0.035** |
| GM-CSF (pg/mL, median [IQR]) | 117.99 [76.68, 205.68] | 33.37 [27.90, 74.03] | **0.027** |
| TPO (pg/mL, median [IQR]) | 1584.33 [1080.27, 2628.55] | 629.55 [415.32, 885.58] | **0.046** |

INCS, intranasal corticosteroids; ELP, eucalyptol, limonene, and pinene enteric capsule; IQR, interquartile range; GM-CSF, granulocyte–macrophage colony-stimulating factor; TPO, thrombopoietin; IL, interleukin; TNF-α, tumor necrosis factor-alpha; G-CSF, granulocyte colony-stimulating factor. ^a^ Mann‒Whitney U test.

**Table S8.** Within group comparison between baseline and week 4/8/12.

|  | ELP + INCS group | | | |  | INCS group | | | |  |
| --- | --- | --- | --- | --- | --- | --- | --- | --- | --- | --- |
|  | Baseline | Week 4 | Week 8 | Week 12 |  | Baseline | Week 4 | Week 8 | Week 12 |  |
| Lund–Kennedy total scores, median (*P* value^a^) | 8.00 | 5.00  (**<0.001^***^**) | 3.00  (**<0.001^***^**) | 3.00  (**<0.001^***^**) |  | 8.00 | 6.00  (**0.003^**^**) | 4.00  (**<0.001^***^**) | 4.00  (**<0.001^***^**) |  |
| Lund–Mackay total scores, median (*P* value^a^) | 17.00 | / | / | 4.50  (**<0.001^***^**) |  | 19.00 | / | / | 6.00  (**<0.001^***^**) |  |
| SNOT-22 total scores, median (*P* value^a^) | 38.00 | 17.00  (**<0.001^***^**) | 10.00  (**<0.001^***^**) | 9.00  (**<0.001^***^**) |  | 37.00 | 13.00  (**<0.001^***^**) | 11.00  (**<0.001^***^**) | 9.00  (**<0.001^***^**) |  |
| Saccharin transit time (STT), median (*P* value^a^) | 360.00 | 405.00  (**0.022^*^**) | 444.50  (**<0.001^***^**) | 449.50  (**0.001^**^**) |  | 400.00 | 589.00  (**0.041^*^**) | 487.00  (**0.026^*^)** | 504.00  (**0.039^*^**) |  |

INCS, intranasal corticosteroids; ELP, pinene enteric capsule; SNOT-22, the 22-Sinonasal Outcomes Test. ^a^ Wilcoxon Signed Rank Test. *P* value, the difference between baseline and week 4/8/12 within each group. * P<0.05, ** P<0.01, *** P<0.001.
